# Regulation of early B cell tolerance checkpoints in humans

**DOI:** 10.1186/1479-5876-9-S2-I11

**Published:** 2011-11-23

**Authors:** Eric Meffre

**Affiliations:** 1 Dept. of Immunobiology, Yale University School of Medicine, New Haven, USA

## 

Understanding the mechanisms that prevent or account for the production of autoreactive B cells may suggest new approaches to control disease and design more specific and sustained therapies.Indeed, an important role for B cells in autoimmune diseases has recently been demonstrated by successful treatment of rheumatoid arthritis (RA), multiple sclerosis (MS), and type 1 diabetes (T1D) patients with anti-CD20 monoclonal antibodies that eliminate B cells. However, the underlying mechanisms by which B cells may promote the development of autoimmune diseases remain poorly understood.Studying B cell tolerance in PID patients allowed us to identify molecules and pathways that regulate the removal of developing autoreactive B cells that is impaired in patients with RA, systemic lupus erythematosus (SLE) and T1D. For instance, alterations in B-cell receptor (BCR) signaling threshold in patients lacking functional BTK, CD19, or molecules mediating Toll-like receptor (TLR) signaling such as IRAK-4, MyD88, and UNC-93B result in a defective central B cell tolerance checkpoint and in the abnormal negative selection of developing autoreactive B cells [1]. In addition, we identified a major and previously unsuspected role for activation-induced cytidinedeaminase (AID), an enzyme required for class switch recombination (CSR) and somatic hypermutation (SHM), in the removal of developing autoreactive B cells in humans [2].

These observations on rare PID patients are very relevant to the development of common autoimmune diseases in that variations in many genes encoding several components of the BCR signaling pathways such as *PTPN22*, seem to be associated with RA, SLE, or T1D.Indeed, the *PTPN22* risk allele, which encodes an R620W variant inducing decreased BCR signaling, is sufficient to alter the removal of developing autoreactive B cells in healthy donors [3]. Thus, decreased BCR signaling favors the development of autoimmunity by failing to induce central B cell tolerance mechanisms after binding to self-antigens and results in the accumulation of large number of autoreactive B cells in the periphery [1,3].In addition, gene array experiments analyzing mature naïve B cells displaying *PTPN22* risk allele(s) revealed that the association strength of *PTPN22* for autoimmunity may not only be due to the impaired removal of autoreactive B cells but also to the upregulation of genes such as *CD40*, *TRAF1*,and *IRF5*, which encode proteins that promote B cell activation and have been identified as susceptibility genes associated with autoimmune diseases [3]. These data demonstrate that early B cell tolerance defects in autoimmunity can result from specific polymorphisms and precede the onset of disease.
